# Evaluation of a Pharmacist-Led Penicillin Allergy Assessment Program and Allergy Delabeling in a Tertiary Care Hospital

**DOI:** 10.1001/jamanetworkopen.2021.9820

**Published:** 2021-05-13

**Authors:** Nicholas A. Turner, Rebekah Wrenn, Christina Sarubbi, Renee Kleris, Patricia L. Lugar, Christine Radojicic, Rebekah W. Moehring, Deverick J. Anderson

**Affiliations:** 1 Division of Infectious Diseases, Duke University Medical Center, Durham, North Carolina; 2 Duke Center for Antimicrobial Stewardship and Infection Prevention, Durham, North Carolina; 3 University of North Carolina, Rex Healthcare, Raleigh; 4 Division of Pulmonary, Allergy and Critical Care, Duke University Medical Center, Durham, North Carolina

## Abstract

**Importance:**

Penicillin allergies are frequently mislabeled, which may contribute to use of less-preferred alternative antibiotics.

**Objective:**

To evaluate a pharmacist-led allergy assessment program’s association with antimicrobial use and clinical outcomes.

**Design, Setting, and Participants:**

A pharmacist-led allergy assessment program was launched in 2 phases (June 1, 2015, and November 2, 2016) at a single-center tertiary referral hospital. The longitudinal cross-sectional study included all study period adult admissions; hospitalwide outcomes were assessed by segmented regression. Individual outcomes were assessed within an embedded propensity score–matched case-control study of inpatients undergoing comprehensive allergy assessment following self-report of penicillin allergy. Analysis occurred from March 1, 2020, to February 29, 2020.

**Exposures:**

The longitudinal study analyzed hospital-level outcomes over 3 periods: preintervention (15 months), phase 1 (structured allergy history alone, 16 months), and phase 2 (comprehensive assessment including penicillin skin testing, 52 months). The case-control study defined cases as individuals undergoing comprehensive allergy assessment.

**Main Outcomes and Measures:**

Hospital-level outcomes included antibiotic days of therapy per 1000 patient-days and hospital-acquired *Clostridioides difficile* infection (CDI) incidence per 10 000 patient-days. Individual outcomes included antibiotic selection, overall survival, and CDI-free survival.

**Results:**

Longitudinal analysis spanned 2014-2020 (median admissions, 46 416 per year; interquartile range [IQR], 46 001-50 091 per year). Hospitalwide, allergy histories were temporally associated with decreased use of nonpenicillin alternative antibiotics (rate ratio, 0.87; 95% CI, 0.79-0.97) and high-CDI-risk antibiotics (rate ratio, 0.91; 95% CI, 0.85-0.98). Penicillin skin testing was temporally associated with lower hospital-acquired CDI rates (rate ratio, 0.61; 95% CI, 0.43-0.86). The embedded case-control study included 272 cases and 819 controls. Median age was 63 years (interquartile range, 51-73 years), 553 (50.7%) patients were women, and 229 (21.0%) patients were Black. Allergy-assessed patients were less likely to receive high-CDI-risk antibiotics at discharge (odds ratio, 0.66; 95% CI, 0.44-0.98). Estimated reductions in mortality (hazard ratio, 0.77; 95% CI, 0.55-1.07) and hospital-acquired CDI risk (hazard ratio, 0.53; 95% CI, 0.18-1.55) were not statistically significant.

**Conclusions and Relevance:**

Pharmacist-led allergy assessments may be associated with reduced high-CDI-risk antibiotic use at both hospitalwide and individual levels. Although individual reductions in mortality and CDI risk did not achieve significance, divergence of survival curves suggest longer-term benefits of allergy delabeling warrant future study.

## Key Points

**Question:**

Are pharmacist-led allergy assessments associated with improved antibiotic selection and reduced *Clostridioides difficile* infection rates?

**Findings:**

In this longitudinal cross-sectional study examining 46 416 admissions, allergy assessments and penicillin skin testing were temporally associated with reduced use of high *Clostridioides difficile* infection–risk antibiotics, and penicillin skin testing was associated with a reduced incidence of hospital-acquired *Clostridioides difficile* infection. In an embedded propensity-matched case-control study including 1091 patients, penicillin skin testing was similarly associated with reduced receipt of high *Clostridioides difficile* infection–risk antibiotics.

**Meaning:**

Pharmacist-led allergy assessments may be a useful tool for reducing high-risk antibiotic use and hospital-acquired *Clostridioides difficile* infection.

## Teaser

This cross-sectional study examines the association of initiation of pharmacist-led assessment of allergy status in patients with self-reported penicillin allergy with antimicrobial use and clinical outcomes in a tertiary care hospital in the US.

## Introduction

Nearly 10% of the US population reports a penicillin allergy. On formal testing, however, fewer than 5% of reported penicillin allergies are confirmed.^1,2^ As a result, many individuals who report penicillin allergy receive alternative antibiotics that are unnecessarily broad-spectrum or less effective than penicillin agents.^3,4,5^ Many nonpenicillin alternative antibiotics confer increased risk for *Clostridioides difficile* infection (CDI) and self-reported penicillin allergy has been identified as an independent risk factor for CDI.^6,7,8,9^

To address outcomes associated with reported penicillin allergies, varying delabeling protocols have been proposed. Effective interventions include pharmacist-led structured questionnaires, penicillin skin testing, and direct oral challenge.^10,11,12,13^ Multiple studies have suggested that penicillin allergy delabeling can decrease the use of nonpreferred antibiotics at institutional and individual levels. Despite promising associations with antimicrobial prescribing practices, however, few studies have assessed relevant clinical outcomes or the relative outcomes of specific interventions.^14^

We conducted a comprehensive evaluation of structured allergy histories and skin testing as a part of antibiotic stewardship efforts. We incorporated hospital-level antimicrobial use, which is relevant to antimicrobial stewardship, and individual risks of CDI and mortality, which are most relevant to patients and clinicians. Because our program was launched in phases, we could independently evaluate structured allergy histories alone or when coupled with penicillin skin testing. The overall objective was to assess the relative association of the study with hospitalwide use of alternative antibiotics, along with any indirect benefits for *C difficile* rates and individual mortality. We hypothesized that comprehensive allergy assessments might reduce the use of high-CDI-risk antibiotics and, thus, the risk of CDI.

## Methods

### Design

We conducted observational analyses of a comprehensive allergy assessment program that was launched in 2 phases. Hospitalwide outcomes were assessed using interrupted time series with 3 periods: preintervention (15 months from March 1, 2014, to May 31, 2015), phase 1 intervention with structured allergy histories (16 months from June 1, 2015, to October 1, 2016), and phase 2 intervention with the addition of penicillin skin testing (52 months from November 2, 2016, to February 29, 2020). We then performed an embedded propensity score-matched case-control study, with cases defined as patients who received a comprehensive allergy assessment (structured history with penicillin skin testing when indicated) during phase 2. Analysis occurred from March 1, 2020, to February 29, 2020. This study was approved by the institutional review board at Duke University Medical Center with waiver of informed consent for collection of data. The waiver was granted because the study was observational, and all data could be gathered in a secure, retrospective manner without requiring any interventions on participants for study purposes. We adhered to the Strengthening the Reporting of Observational Studies in Epidemiology (STROBE) reporting guideline throughout.

### Hospitalwide Longitudinal Analysis

All adults admitted to Duke University Medical Center from March 1, 2014, through February 29, 2020, were included in the longitudinal analysis. No patients were excluded from this analysis. Preintervention management of reported penicillin allergy was left to the discretion of treating physicians. Phase 1 of the allergy assessment program began June 1, 2015, with structured allergy histories conducted by pharmacists and pharmacy technicians using a previously validated flowchart.^15^ Phase 2 began November 2, 2016, with the addition of penicillin skin testing. Allergists (R.K., P.L.L., and C.R.) trained stewardship pharmacists to administer and interpret penicillin skin test results. Allergy evaluation could be obtained 2 ways: any physician could request penicillin allergy evaluation or stewardship pharmacists conducting audit and feedback rounds could suggest evaluation. Stewardship Allergy Assessment Team (StAAT) members documented their assessment in the patient’s medical record and updated the allergy tab as appropriate. Patients were provided a card to communicate assessment results with outpatient clinicians. In addition, a best-practice alert was created to notify electronic medical record users and StAAT if an allergy label was restored after it had been delabeled.

Monthly inpatient antibiotic use, hospital-acquired CDI incidence rate, and hospital census data were collected from computerized medication administration records and an institutional microbiology surveillance database.

Hospital-level outcomes included narrow-spectrum β-lactam antibiotic use, high-CDI-risk antibiotic use, nonpenicillin alternative antibiotic use, and hospital-acquired CDI rates. High-CDI-risk antibiotic and narrow-spectrum β-lactam antibiotics were defined in accordance with existing National Healthcare Safety Network definitions.^16^ Complete antibiotic classifications are provided in eTable 1 in the Supplement. We constructed an additional class of nonpenicillin alternative antibiotics frequently used in the setting of penicillin allergy but considered less desirable owing to risk for adverse effects (aminoglycosides, fluoroquinolones, and clindamycin), cost (aztreonam), or spectrum (aztreonam). Monthly antibiotic use rates are reported as days of therapy per thousand days present. Hospital-associated CDI was defined as positive results of a *C difficile* stool polymerase chain reaction test obtained any time after hospital day 3, up to 28 days after discharge, combining National Healthcare Safety Network definitions for hospital-onset and health care–associated CDI. Monthly hospital-acquired CDI rates were calculated per 10 000 patient-days present, limited to the time from January 1, 2015, to February 29, 2020, based on the availability of consistent National Healthcare Safety Network census data.

### Embedded Individual Case-Control Analysis

The case-control population included adults (age ≥18 years) with self-reported penicillin allergy admitted to Duke University Hospital between November 1, 2016, and February 29, 2020. Patients were excluded if admitted only for observation (eg, ≤48 hours) (n = 2658), died within the first 72 hours after admission (n = 45), or were missing encounter information (n = 4). Cases included any patient for whom a penicillin allergy assessment consult was completed. Controls included any patients who did not receive an allergy assessment consult.

The case-control population was identified by querying the Duke Enterprise Data Unified Content Explorer for adults with self-reported penicillin allergy admitted during the study period.^17^ Patients assessed by StAAT were identified by cross-referencing a consult database kept in RedCap. Patient-specific covariates were identified by query of the electronic medical record. Race reflects patients’ self-reported race. Comorbidities were assigned according to *International Statistical Classification of Diseases, 10th Revision* code groupings.^18^ Data were verified by manual review of 10 randomly sampled medical records for each query component. Individual outcomes of interest included antibiotic selection, mortality, and hospital-acquired CDI-free survival followed up to 28 days after hospital discharge.

### Statistical Analysis

Hospitalwide antibiotic use was analyzed by interrupted time series with a negative binomial model counting days of therapy. Patient-days present served as an offset term to account for variations in hospital census over time. Separate models were constructed for each antibiotic class of interest. Each antibiotic use model included time, intervention, and a time after intervention term. Intervention covariates were used to assess for level (intercept) changes, and time after intervention terms were used to assess for slope (trend) changes.^19^ Each model was assessed for autocorrelation by the Breusch-Godfrey test. Without standardized methods for power analysis of interrupted time series regression, no formal power calculations were conducted.

We used propensity score matching to address selection bias among allergy-assessed cases.^20^ Individual propensities for assessment were estimated using a logistic regression model with 35 covariates thought to influence selection for assessment or CDI risk (eTable 2 in the Supplement). Covariates included age, sex, race, empirical antibiotic receipt, infection type, infectious diseases consultation, treating service, and comorbidities according to the Charlson Comorbidity Index.^21^ Assessed patients (cases) were matched 1:3 with unassessed controls using an optimal matching algorithm.^22,23,24^ Balance of covariates was assessed using standardized mean differences, with less than 0.1 considered to indicate balance.

Individual antibiotic selection was compared between cases and controls using logistic regression. To account for varying time at risk, we used a time-to-event analysis strategy for individual outcomes in mortality and CDI. Separate Cox proportional hazard models were developed for overall and CDI-free survival. The proportional hazards assumption was verified for each model by inspection of Schoenfeld residual plots. To account for violation of the independence assumption in matched data, we stratified models by propensity score quantiles. Within the CDI-free survival model, death due to a non–CDI-related cause was considered a censoring event.

Throughout both phases of the study, *P* < .05 was considered statistically significant; all tests were 2-sided. All data management, propensity matching, and statistical analyses were conducted using R, version 3.5.3.^25^

## Results

Longitudinal analysis spanned 6 years (2014-2020) with a median of 46 416 admissions (interquartile range, 46 001-50 091 admissions) per year. A total of 194 803 of 1 324 839 (14.7%) adults who were admitted reported a penicillin allergy. The embedded propensity-matched cohort included 272 cases and 819 controls. Median age was 63 years (interquartile range, 51-73 years), 553 (50.7%) patients were women, 538 (49.3%) patients were men, and 229 (21.0%) patients were Black.

### Allergy Evaluation Program

Throughout the study, pharmacists conducted approximately 1500 structured allergy histories per month (mean [SD], 22.4 [8.3]) and StAAT performed 193 penicillin skin tests (mean [SD], 3.7 [2.6] per month). During phase 2, a total of 11 581 patients with self-reported penicillin allergy were admitted; of these, 273 (2.4%) underwent assessment by StAAT. Among patients assessed by StAAT, 47 (17.2%) were considered to have no penicillin allergy by detailed allergy history alone, 193 (70.7%) proceeded to penicillin skin testing, 8 (2.9%) underwent direct oral challenge, 3 (1.1%) could not be tested owing to interfering medications (prednisone or antihistamines), 6 (2.2%) deferred testing to the outpatient setting, 1 (0.4%) declined testing, and 15 (5.5%) lacked a documented reason for incomplete testing. Among 193 patients who underwent skin testing, 187 (96.9%) tested negative, 4 (2.1%) tested positive, and the results for 2 (1.0%) were indeterminate. Among the cohort that was delabeled, no patients were documented to have subsequent reactions to penicillin-based antibiotic therapy.

### Hospitalwide Outcomes

**Figure 1.**
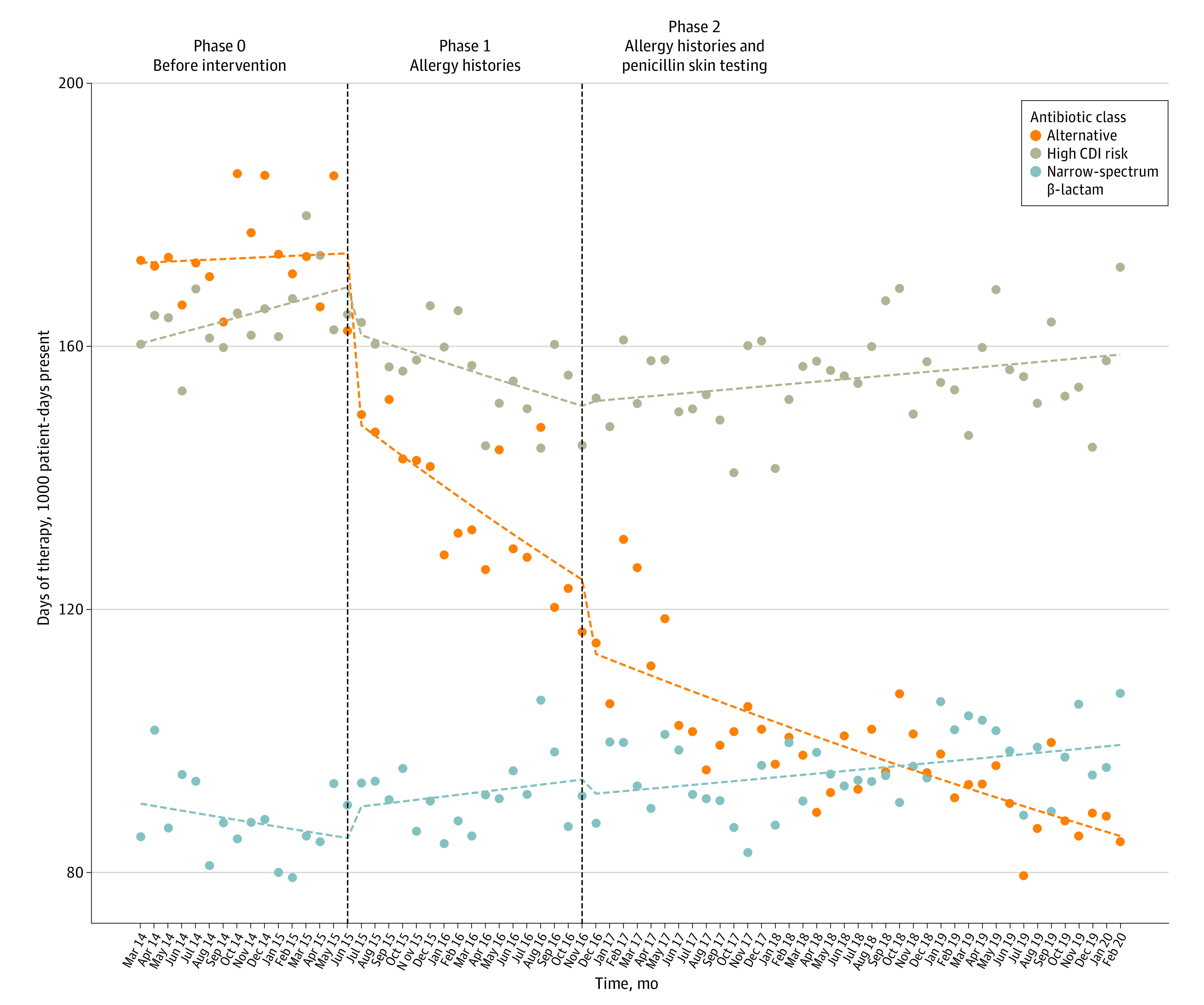
Interrupted Time Series Analysis of Antibiotic Use by Antibiotic Class Vertical dashed lines represent transitions between intervention phases. Points represent actual antibiotic use in days of therapy per thousand patient-days present. Horizontal dashed line represents modeled results from interrupted time series regression.

Figure 1 summarizes hospitalwide antibiotic use. Prior to phase 1 of the study, the rates of narrow-spectrum β-lactam agent use were stable (rate ratio, 0.95; 95% CI, 0.89-1.02). Although use of narrow-spectrum β-lactam antibiotics modestly increased beyond the baseline period, neither phase 1 (rate ratio, 1.08; 95% CI, 0.98-1.20) nor phase 2 (rate ratio, 1.00; 95% CI, 0.99-1.01) interventions were significantly associated with any trend change. Phase 1 was temporally associated with a downward trend in high-CDI-risk antibiotic use (use rate ratio, 0.91; 95% CI, 0.85-0.98), but phase 2 was not (use rate ratio, 1.07; 95% CI, 1.01-1.12). Complete effect size estimates are included in eTable 3 in the Supplement. Sensitivity analysis for outcomes associated with an enhanced fluoroquinolone black box warning released in July 2016 did not significantly alter the estimated effect size of allergy assessments (eFigure 1 in the Supplement). Allergy assessments were temporally associated with level (rate ratio, 0.85; 95% CI, 0.79-0.92) and trend (rate ratio, 0.87; 95% CI, 0.79-0.97) reductions in nonpenicillin alternative antibiotic use. The addition of penicillin skin testing was limited to a modest level reduction in alternative antibiotic use (rate ratio, 0.92; 95% CI, 0.86-0.98).

Analyses of individual antibiotic class uses are included in eFigure 2 in the Supplement. Most of the observed decreases in alternative and high-CDI-risk antibiotic use were attributable to decreases in fluoroquinolone, monobactam, carbapenem, and lincosamide use—all of which were temporally associated with the phase 1 study. Penicillin use showed a modest increase.

**Figure 2.**
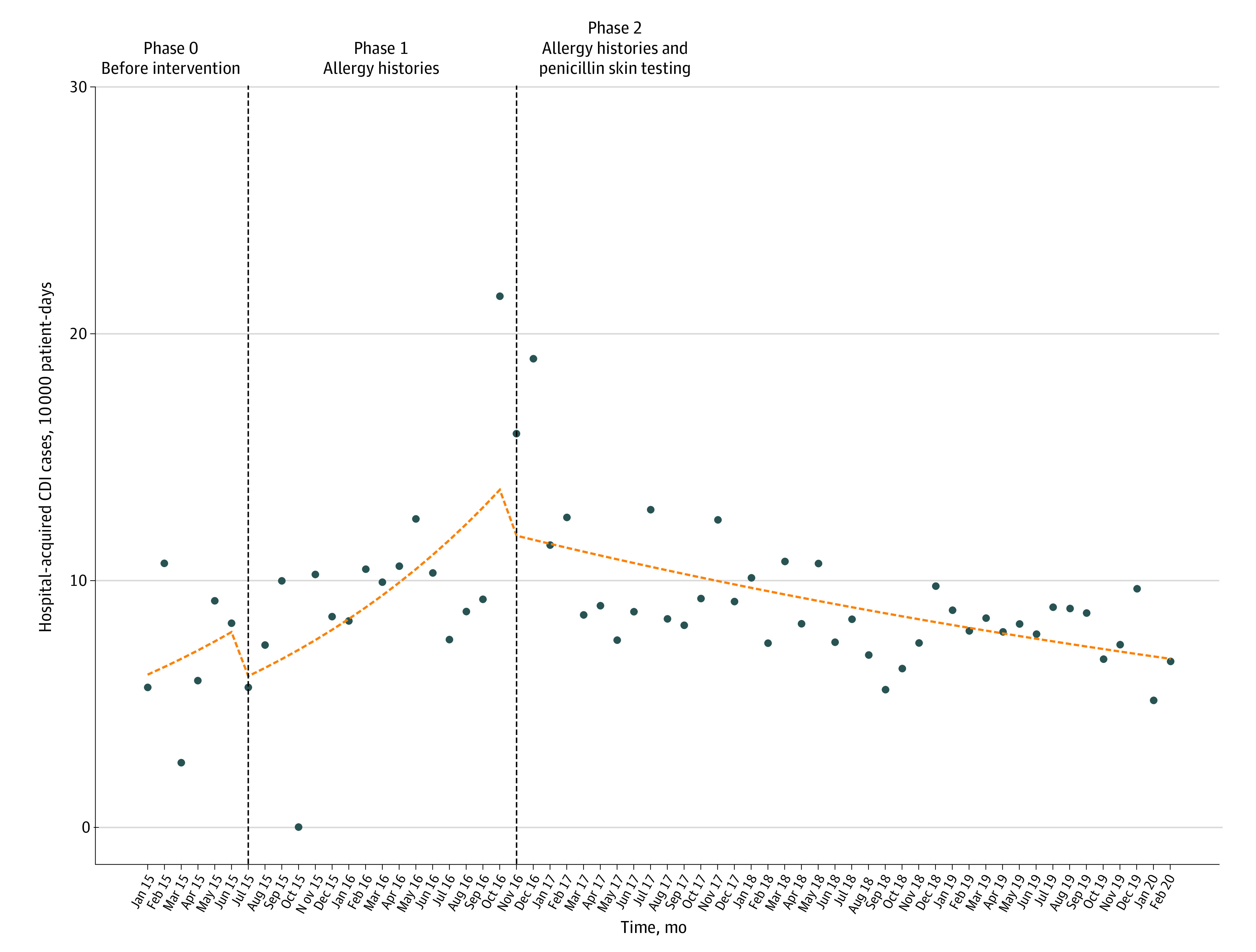
Interrupted Time Series Analysis of Hospital-Acquired *Clostridioides difficile* Infection (CDI) Rates Vertical dashed lines represent transitions between intervention phases. Points represent actual hospital-acquired CDI incidence rates per 10 000 patient-days present. Horizontal dashed line represents modeled results from interrupted time series regression.

Neither phase 1 nor phase 2 findings were associated with level changes in hospital-acquired CDI rates. Phase 2 results were temporally associated with a downward trend in hospital-acquired CDI rates (incident rate ratio, 0.61; 95% CI, 0.43-0.86) (Figure 2). Effect size estimates are presented in eTable 4 in the Supplement. An increase in the incidence of hospital-acquired CDI occurred from October 1 to December 31, 2016. Because an episode of increased incidence between phase 1 and phase 2 risks confounding by regression to the mean, we conducted a post hoc sensitivity analysis excluding these months as potential outliers. The estimated reduction in hospital-acquired CDI associated with phase 2 interventions decreased but remained statistically significant (incident rate ratio, 0.96; 95% CI, 0.93-0.99; *P* = .01) (eFigure 3 in the Supplement).

### Individual Outcomes

Propensity scores were calculated for all 11 581 patients reporting penicillin allergy, with a distribution summarized in eFigure 4 in the Supplement. Propensity scores ranged from less than 0.01 to 0.82. The propensity score model’s area under the receiver operating characteristic curve was 0.90 (bootstrap 95% CI, 0.89-0.92).

**Figure 3.**
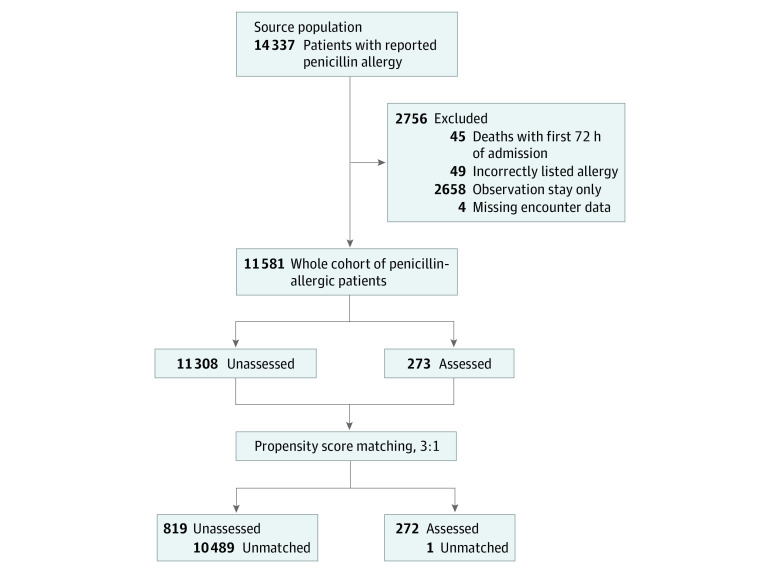
Cohort of Patients With Self-reported Penicillin Allergy Remaining Unassessed, Matched Unassessed, and Assessed

The matched analysis included 272 patients assessed in phase 2 and 819 unassessed controls (Figure 3). One assessed patient could not be matched. Within the overall sampling population, multiple imbalances were noted, consistent with suspected selection bias in testing. After matching, covariates were well balanced (Table), achieving standardized mean differences of less than 0.10 for every variable evaluated.

**Table.** Baseline Characteristics of Overall Sampling Population and Matched Controls

Variable	Propensity score matching						
	Before matching			After matching			Standardized mean difference^a^
	No. (%)		*P* value	No. (%)		*P* value	
	Unevaluated	Evaluated		Unevaluated	Evaluated		
Patients	11 308 (97.6)	273 (2.4)		819 (75.0)	272 (25.0)		
Age, median (IQR), y	66 (54-76)	63 (49-73)	.02	63 (51-73)	63 (49-73)	.72	0.03
Sex							0.03
Female	7320 (64.7)	135 (49.5)	<.01	418 (51.0)	135 (49.6)	.74	
Male	3988 (35.3)	138 (50.5)		401 (49.0)	137 (50.4)		
Race							0.02
Black	3018 (26.7)	57 (20.9)	.06	172 (21.0)	57 (21.0)	.96	
White	7764 (68.7)	206 (75.5)		620 (75.7)	205 (75.4)		
Other	526 (4.7)	10 (3.7)		27 (3.3)	10 (3.7)		
Treating service							0.07
Medicine	6672 (59.0)	162 (59.3)	.08	508 (62.0)	161 (59.2)	.57	
Oncology	483 (4.3)	19 (7.0)		62 (7.6)	19 (7.0)		
Surgery	4153 (36.7)	92 (33.7)		249 (30.4)	92 (33.8)		
Infection type							
Pneumonia	895 (7.9)	35 (12.8)	<.01	121 (14.8)	35 (12.9)	.50	0.06
UTI	226 (2.0)	11 (4.0)	.03	34 (4.2)	11 (4.0)	.99	0.01
Endocarditis	58 (0.5)	13 (4.8)	<.01	28 (3.4)	13 (4.8)	.40	0.07
Bacteremia	1112 (9.8)	97 (35.5)	<.01	284 (34.7)	97 (35.7)	.82	0.02
Osteomyelitis	218 (1.9)	33 (12.1)	<.01	92 (11.2)	33 (12.1)	.77	0.03
Intra-abdominal	700 (6.2)	22 (8.1)	.26	74 (9.0)	22 (8.1)	.72	0.03
ABSSSI	662 (5.9)	59 (21.6)	<.01	158 (19.3)	59 (21.7)	.44	0.06
Comorbidities							
Cancer	1863 (16.5)	62 (22.7)	<.01	185 (22.6)	62 (22.8)	.99	0.01
Congestive heart failure	1973 (17.4)	69 (25.3)	<.01	181 (22.1)	68 (25.0)	.37	0.07
Chronic kidney disease	1978 (17.5)	65 (23.8)	<.01	184 (22.5)	65 (23.9)	.69	0.03
Connective tissue diseases	487 (4.3)	11 (4.0)	.94	34 (4.2)	11 (4.0)	.99	0.01
Cerebrovascular disease;	1249 (11.0)	47 (17.2)	<.01	120 (14.7)	47 (17.3)	.34	0.07
Dementia	269 (2.4)	8 (2.9)	.70	21 (2.6)	8 (2.9)	.91	0.02
Diabetes	2736 (24.2)	78 (28.6)	.11	227 (27.7)	78 (28.7)	.82	0.02
HIV	78 (0.7)	4 (1.5)	.25	15 (1.8)	4 (1.5)	.90	0.03
Liver	782 (6.9)	34 (12.5)	<.01	115 (14.0)	34 (12.5)	.59	0.05
Pulmonary	2697 (23.9)	80 (29.3)	.04	228 (27.8)	79 (29.0)	.76	0.03
Vascular	1448 (12.8)	56 (20.5)	<.01	146 (17.8)	56 (2.6)	.36	0.07
Transplant	388 (3.4)	16 (5.9)	.05	57 (7.0)	16 (5.9)	.63	0.04
Modified CCI score, median (IQR)	3.5 (1.7-6.0)	4.0 (2.0-7.3)	<.01	4.0 (1.9-7.2)	4.0 (2.0-7.3)	.44	0.05
Encounter specific							
Infectious diseases consult	1135 (10.0)	175 (64.1)	<.01	514 (62.8)	175 (64.3)	.69	0.03
Shock	2050 (18.1)	60 (22.0)	.12	182 (22.2)	60 (22.1)	.99	0.01
Length of stay, median (IQR), d	5.3 (3.9-8.3)	8.5 (5.2-14.7)	.31	8.1 (5.4-14.9)	8.5 (5.2-14.7)	.87	0.01
Alternative antibiotics^b^	2404 (21.3)	150 (54.9)	<.01	436 (53.2)	150 (55.1)	.63	0.04
Antibiotics >72 h	1998 (17.7)	198 (72.5)	<.01	594 (72.5)	197 (72.4)	.99	0.01

Abbreviations: ABSSSI, acute bacterial skin and skin structure infection; CCI, Charlson Comorbidity Index; HIV, human immunodeficiency virus; IQR, interquartile range; UTI, urinary tract infection.

^a^ Values less than 0.1 are considered optimal for matching purposes.

^b^ Defined as fluoroquinolones, lincosamides, and monobactams.

Comparing assessed patients with matched controls, comprehensive penicillin allergy assessment was associated with an increased likelihood of β-lactam (odds ratio [OR], 12.3; 95% CI, 9.0-17.0) or early-generation cephalosporin (OR, 1.46; 95% CI, 1.05-2.02) receipt before discharge. Penicillin allergy assessment was associated with an increased likelihood of β-lactam receipt upon discharge (OR, 7.19; 95% CI, 4.03-13.48). Despite more frequent inpatient receipt of high-CDI-risk antibiotics, assessed patients had a decreased likelihood of high-CDI-risk antibiotic receipt on discharge (OR, 0.66; 95% CI, 0.44-0.98) (eTable 5 in the Supplement). Days of therapy by antibiotic class showed similar trends (eTable 6 in the Supplement).

**Figure 4.**
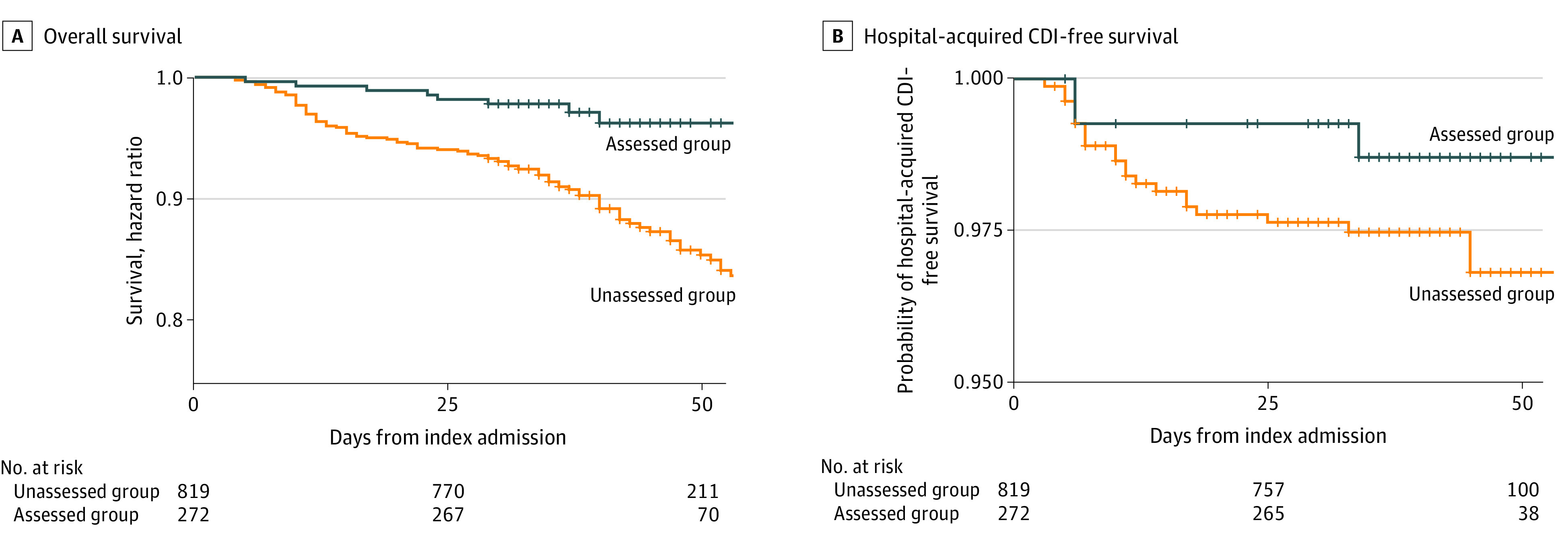
Overall and Hospital-Acquired *Clostridioides difficile* Infection (CDI)-Free Survival Curves A, Overall survival, stratified by penicillin allergy assessment. B, Hospital-acquired CDI-free survival, stratified by penicillin allergy assessment.

Within clinical outcomes, estimated reductions in mortality after penicillin allergy assessment (hazard ratio, 0.77; 95% CI, 0.55-1.07) and hospital-acquired CDI risk (hazard ratio, 0.53; 95% C 0.18-1.55) were not statistically significant (Figure 4). Accompanying effect size estimates are presented in eTable 7 in the Supplement.

## Discussion

Penicillin allergy delabeling improves antibiotic selection, with potential benefit at both individual and institutional levels. Despite multiple studies reporting various allergy delabeling strategies on antibiotic selection, few have assessed clinical outcomes in parallel at both hospital and individual levels.^3,6,14,26,27,28^ This study’s construction—with both hospital-level longitudinal analysis and an embedded propensity-matched case-control study—permitted comprehensive assessment of a unique pharmacist-led allergy evaluation program.

Consistent with earlier studies, we noted that formal allergy evaluations were associated with statistically significant reductions in alternative and high-CDI-risk antibiotic use over time.^4,5,6^ Although only randomized clinical trials can prove causation, our study addressed key components that can be used in causal assessment. The observed reduction in high-CDI-risk antibiotic use provides a plausible mechanism, and interrupted time series offers a useful quasi-experimental design for assessing temporal correlation while adjusting for preexisting trends.

Mirroring hospital-level associations, our embedded propensity matched case-control study found similar benefits for individuals undergoing penicillin allergy assessment. Despite more frequent initial receipt of high-CDI-risk antibiotics, patients who underwent skin testing had a decreased likelihood of receiving high-CDI-risk antibiotics on discharge. Instead, these patients were more likely to receive a β-lactam at discharge, suggesting allergy assessment influenced the selection of definitive therapy.

The stronger association between allergy assessment on discharge and antibiotic selection highlights a consideration for assessing the use of inpatient allergy evaluation. Inpatients with allergy labels are likely to receive empirical antibiotic therapy while awaiting allergy evaluation. Consequently, any effect of allergy assessment on initial antibiotic exposure is inherently limited. Instead, allergy assessment is more likely to be associated with antibiotic selection when definitive therapy is chosen at hospital discharge or subsequent encounters. Consistent with a possibly stronger influence on definitive therapy, we observed a greater association with discharge antibiotic selection than inpatient therapy.

The potentially delayed benefits associated with allergy delabeling are also suggested by the overall and CDI-free survival curves. Although neither mortality nor CDI rates differed significantly between assessed and unassessed patients, 2 factors may have influenced our results. First, our sample sizes were small, increasing uncertainty in effect size estimates. Second, our follow-up period was limited. The 28-day follow-up period was selected to ensure CDI events were related to the hospitalization of interest. Although longer follow-up increases risk for confounding from subsequent unmeasured exposures, visual inspection of CDI-free survival curves suggests that CDI risk continues to diverge over time. Consequently, penicillin allergy assessment may confer accruing benefit over time, perhaps mediated by continued association between allergy delabeling and subsequent antibiotic treatment. A similar potential for delayed benefit might also explain why penicillin allergy assessment was associated with a slope change in CDI rate rather than immediate-level changes. Any hospitalwide association would require time to accrue a sufficient number of patients no longer considered to have a penicillin allergy such that the outcome becomes measurable. Overrepresentation of patients with reported penicillin allergy among frequently admitted patients may explain part of the seemingly outsized association between allergy testing and hospital level outcomes. Future studies with longer follow-up are needed to further examine the potential for ongoing accrual of benefit for both hospitals and individuals.

The unique pharmacist-led protocol has the potential to expand access to delabeling opportunities while also being integrated within existing antibiotic stewardship infrastructure. Although not directly assessed in this study, the integration between allergy delabeling and stewardship pharmacists may have been associated with increased delabeling. In addition, the observed reduction in high-CDI-risk antibiotic use with structured allergy assessments alone is encouraging. Given the smaller number of patients no longer considered allergic following penicillin skin testing and greater resources required, structured allergy histories alone may still represent a useful option for sites unable to offer comprehensive assessments.

### Limitations

Our study carries several limitations. Penicillin skin testing was used in a small number of patients. Although there were no discrete hospitalwide interventions amenable to segmented regression analysis during the study period, efforts to improve antibiotic stewardship and reduce hospital-acquired CDI were ongoing within departments and may confound our results. We assessed for an association between enhanced black box labeling of fluoroquinolones; however, it was not possible to adjust for every potential confounder over time (eg, other stewardship efforts, formulary changes, and drug shortages). In addition, the outcome of penicillin skin testing on hospital-acquired CDI is confounded by an increase in hospital-acquired CDI incidence near the end of phase 1, which risks misattributing benefit from what may actually be regression to the mean. On sensitivity analysis, penicillin skin testing remained temporally associated with lower hospital-acquired CDI incidence, although the effect size was markedly reduced.

Among individual outcomes, there is inherent risk of selection bias. Perceived benefit might have differed for individuals anticipated to survive longer, those with a specific indication for which β-lactam antimicrobials are preferred, or those likely to receive repeated courses of antibiotics. Despite extensive efforts to address selection bias, including propensity matching, residual bias may persist. As a further limitation, CDI cases and outpatient antibiotic prescriptions were assessed by electronic query, which risks missing cases or prescriptions occurring outside of our health care system. In addition, the interventions described herein were undertaken within a tertiary medical center using a team of specially trained stewardship pharmacists. The study results may not be generalizable to sites without such resources.

## Conclusions

The findings of this study suggest that a comprehensive, pharmacist-led penicillin allergy assessment program may be associated with reduced alternative and high-CDI-risk antibiotic use at both the hospital and individual levels. Although inherent limitations of observational studies prevent us from attributing hospital-acquired CDI reductions to allergy delabeling, our results suggest that allergy delabeling may be a useful component of broader antibiotic stewardship interventions. Small sample size and follow-up period limit the assessment of individual benefits for CDI-free survival; however, ongoing divergence in survival curves suggests the potential for accrued benefits over time. Collectively, a comprehensive, pharmacist-led allergy assessment program improves antibiotic selection, which may in turn lessen CDI risk for both hospitals and individuals over time. Confirming the potential benefits of comprehensive allergy assessment will require longer follow-up and prospective evaluation.

## Notes

Supplement. **eTable 1.** Antibiotic Classification **eTable 2.** Summary of Covariates Used in Developing Logistic Regression Model Predictive of Penicillin Skin Testing **eTable 3.** Interrupted Time Series Analysis of Antibiotic Use by Antibiotic Class **eFigure 1.** Sensitivity Analysis of Interrupted Time Series Analysis of High CDI Risk Antibiotics **eFigure 2.** Interrupted Time Series Analysis by Individual Antibiotic Classes **eTable 4.** Interrupted Time Series Analysis of Hospital-Associated *C difficile* Rates **eFigure 3.** Sensitivity Analysis for Potential Outlier Effect of October 2016-December 2016 HA-CDI Rates **eFigure 4.** Distribution of Propensity Scores **eTable 5.** Inpatient and Discharge Antibiotic Selection Analyzed by Penicillin Allergy Assessment Status **eTable 6.** Inpatient and Outpatient Days of Therapy by Antibiotic and Penicillin Skin Testing Status **eTable 7.** Cox Proportional Hazard Estimates
