# Supplementary material for: Evaluation of a Pharmacist-Led Penicillin Allergy Assessment Program and Allergy Delabeling in a Tertiary Care Hospital
Source: JAMA Netw Open. 2021 May 13;4(5):e219820. doi: 10.1001/jamanetworkopen.2021.9820 (PMC8120333; doi:10.1001/jamanetworkopen.2021.9820)
Supplement: Supplement. — eTable 1. Antibiotic Classification eTable 2. Summary of Covariates Used in Developing Logistic Regression Model Predictive of Penicillin Skin Testing eTable 3. Interrupted Time Series Analysis of Antibiotic Use by Antibiotic Class eFigure 1. Sensitivity Analysis of Interrupted Time Series Analysis of High CDI Risk Antibiotics eFigure 2. Interrupted Time Series Analysis by Individual Antibiotic Classes eTable 4. Interrupted Time Series Analysis of Hospital-Associated C difficile Rates eFigure 3. Sensitivity Analysis for Potential Outlier Effect of October 2016-December 2016 HA-CDI Rates eFigure 4. Distribution of Propensity Scores eTable 5. Inpatient and Discharge Antibiotic Selection Analyzed by Penicillin Allergy Assessment Status eTable 6. Inpatient and Outpatient Days of Therapy by Antibiotic and Penicillin Skin Testing Status eTable 7. Cox Proportional Hazard Estimates [file jamanetwopen-e219820-s001.pdf]

Supplementary Online Content

Turner NA, Wrenn R, Sarubbi C, et al. Evaluation of a pharmacist-led penicillin allergy assessment program and allergy delabeling in a tertiary care hospital. *JAMA Netw Open*. 2021;4(5):e219820. doi:10.1001/jamanetworkopen.2021.9820

eTable 1. Antibiotic Classification

eTable 2. Summary of Covariates Used in Developing Logistic Regression Model Predictive of Penicillin Skin Testing

eTable 3. Interrupted Time Series Analysis of Antibiotic Use by Antibiotic Class

eFigure 1. Sensitivity Analysis of Interrupted Time Series Analysis of High CDI Risk Antibiotics

eFigure 2. Interrupted Time Series Analysis by Individual Antibiotic Classes

eTable 4. Interrupted Time Series Analysis of Hospital-Associated *C difficile* Rates

eFigure 3. Sensitivity Analysis for Potential Outlier Effect of October 2016-December 2016 HA-CDI Rates

eFigure 4. Distribution of Propensity Scores

eTable 5. Inpatient and Discharge Antibiotic Selection Analyzed by Penicillin Allergy Assessment Status

eTable 6. Inpatient and Outpatient Days of Therapy by Antibiotic and Penicillin Skin Testing Status

eTable 7. Cox Proportional Hazard Estimates

This supplementary material has been provided by the authors to give readers additional information about their work.

© 2021 Turner NA et al. *JAMA Network Open.*

eTable 1: Antibiotic Classification

Antibiotic Grouping	Antibiotics Included
Penicillins	Amoxicillin, amoxicillin with clavulanate, ampicillin, ampicillin with sulbactam, dicloxacillin, nafcillin, oxacillin, penicillin, piperacillin with tazobactam, ticarcillin, ticarcillin with clavulanate
Early generation cephalosporin (1st-2nd)	Cefaclor, cefadroxil, cefazolin, cefotetan, cefoxitin, cefprozil, cefuroxime, cephalexin
Late generation cephalosporin (3rd-4th)	Cefdinir, cefditoren, cefepime, cefixime, cefotaxime, cefpodoxime, ceftaroline, ceftazidime, ceftazidime with avibactam, ceftibuten, ceftizoxime, ceftolozane with tazobactam, ceftriaxone
Fluoroquinolones	Ciprofloxacin, gemifloxacin, moxifloxacin, levofloxacin
Lincosamide	Clindamycin
Monobactam	Aztreonam
Gram positive agents	Vancomycin, daptomycin, dalbavancin, linezolid, tedizolid, oritavancin
Carbapenem	Doripenem, ertapenem, imipenem, meropenem
High risk for *C. difficile* infection	Cefdinir, cefepime, cefixime, cefotaxime, ceftazidime, ceftriaxone, ciprofloxacin, clindamycin, gemifloxacin, levofloxacin, moxifloxacin
Macrolides	Azithromycin, clarithromycin
Sulfa	Trimethoprim-sulfamethoxazole
Tetracyclines	Doxycycline, tetracycline
Aminogylcosides	Amikacin, gentamicin, tobramycin, streptomycin
Other	Metronidazole, nitrofurantoin
Non-penicillin Alternative Agents	Aztreonam, ciprofloxacin, moxifloxacin, levofloxacin, clindamycin, amikacin, gemifloxacin, gentamicin, tobramycin, streptomycin
Narrow spectrum beta-lactam agents	Amoxicillin, amoxicillin-clavulanate, ampicillin, ampicillin-sulbactam, cefadroxil, cefazolin, cefotetan, cefoxitin, cephalexin, dicloxacillin, nafcillin, oxacillin, penicillin

© 2021 Turner NA et al. *JAMA Network Open.*

eTable 2: Summary of covariates used in developing logistic regression model predictive of penicillin skin testing.

	Multivariate Logistic Regression Modeling in Whole Cohort			
	For Penicillin Skin Testing		For HCFA CDI	
Variables	OR (95% CI)	P value	OR (95% CI)	P value
Penicillin skin tested	--	--	0.48 (0.14-1.21)	0.17
Age, per decade[a]	0.97 (0.88-1.08)	0.63	1.17 (0.99-1.38)	0.06
Female	Ref	--	Ref	--
Male	1.31 (1.00-1.72)	0.05	0.67 (0.44-1.01)	0.06
Race				
Black	Ref	--	Ref	--
White	1.30 (0.94-1.82)	0.12	0.92 (0.68-1.27)	0.90
Other	0.78 (0.35-1.55)	0.49	0.84 (0.37-1.67)	0.79
Treating Service				
Medicine	Ref	--	Ref	--
Oncology	0.66 (0.35-1.19)	0.18	1.45 (0.59-3.31)	0.40
Surgery	0.79 (0.58-1.08)	0.14	1.13 (0.73-1.74)	0.57
Infection Type				
Pneumonia	1.00 (0.65-1.51)	0.99	1.18 (0.65-2.03)	0.56
UTI	2.46 (1.17-4.67)	0.01	1.12 (0.27-3.10)	0.85
Endocarditis	1.70 (0.79-3.46)	0.16	0.59 (0.03-2.97)	0.62
Bacteremia	1.40 (1.02-1.92)	0.04	0.91 (0.52-1.54)	0.74
Osteomyelitis	1.47 (0.92-2.31)	0.10	0.35 (0.05-1.19)	0.16
Intra-abdominal	1.17 (0.70-1.87)	0.53	1.52 (0.80-2.69)	0.17
ABSSSI	1.70 (1.18-2.41)	<0.01	0.93 (0.42-1.82)	0.85
Co-morbidities				
Cancer	1.22 (0.77-1.89)	0.38	0.84 (0.42-1.60)	0.60
CHF	1.43 (0.96-2.12)	0.08	0.87 (0.48-1.51)	0.62
CKD	0.81 (0.52-1.24)	0.33	1.55 (0.86-2.75)	0.14
CTD	0.92 (0.44-1.74)	0.81	0.95 (0.33-2.18)	0.91
CVA	1.71 (1.15-2.52)	<0.01	0.98 (0.51-1.75)	0.96
Dementia	2.00 (0.84-4.18)	0.09	1.85 (0.64-4.40)	0.20
Diabetes	0.93 (0.65-1.31)	0.67	0.78 (0.45-1.31)	0.35
HIV	0.34 (0.09-1.01)	0.07	1.98 (0.39-7.60)	0.35
Liver	1.38 (0.86-2.16)	0.17	1.53 (0.76-2.92)	0.21
Pulmonary	1.42 (1.02-1.95)	0.04	0.93 (0.56-1.48)	0.75
Vascular	1.22 (0.83-1.77)	0.30	1.06 (0.58-1.83)	0.85
Transplant	0.67 (0.36-1.20)	0.20	0.97 (0.40-2.06)	0.93
mCCMI[a]	1.04 (0.95-1.12)	0.40	1.01 (0.89-1.13)	0.84
Encounter Specific				
ID consult	8.14 (5.93-11.22)	<0.01	2.53 (1.52-4.13)	<0.01
Shock	0.70 (0.50-0.96)	0.03	0.92 (0.57-1.44)	0.72
Length of stay[a]	0.99 (0.98-0.99)	0.02	1.02 (1.01-1.03)	<0.01
Alternative antibiotics	2.13 (1.57-2.89)	<0.01	0.94 (0.59-1.49)	0.81
Antibiotics >72h	4.90 (3.49-6.92)	<0.01	3.48 (2.15-5.56)	<0.01

© 2021 Turner NA et al. *JAMA Network Open.*

eTable 3: Interrupted time series analysis of antibiotic use by antibiotic class – Effect Estimates Accompanying Figure 1

Model	Use Rate Ratio (95% CI)	p-value
**Non-penicillin Alternative**[a]		
Pre-intervention	1.01 (0.94-1.08)	0.86
Phase 1, level change	0.85 (0.79-0.92)	<0.01
Phase 1, slope change	0.87 (0.79-0.97)	0.01
Phase 2, level change	0.92 (0.86-0.98)	0.01
Phase 2, slope change	1.04 (0.97-1.12)	0.28
**High CDI risk antibiotics**[b]		
Pre-intervention	1.04 (0.99-1.10)	0.10
Phase 1, level change	0.95 (0.90-1.01)	0.09
Phase 1, slope change	0.91 (0.85-0.98)	0.01
Phase 2, level change	1.00 (0.96-1.05)	0.87
Phase 2, slope change	1.07 (1.01-1.12)	0.01
**Narrow Spectrum Beta Lactam**[b]		
Pre-intervention	0.95 (0.89-1.02)	0.20
Phase 1, level change	1.06 (0.98-1.15)	0.14
Phase 1, slope change	1.08 (0.98-1.20)	0.12
Phase 2, level change	0.98 (0.91-1.04)	0.47
Phase 2, slope change	1.00 (0.99-1.01)	0.82

[a]Clindamycin, fluoroquinolones, aztreonam, aminoglycosides

[b]Clindamycin, fluoroquinolones, 3rd and 4th generation cephalosporins

[c]Amoxicillin±clavulanate, ampicillin±sulbactam, nafcillin, oxacillin, penicillin, and 1st or 2nd generation cephalosporins

© 2021 Turner NA et al. *JAMA Network Open.*

eFigure 1: Sensitivity analysis of interrupted time series analysis of high CDI risk antibiotics.

Sensitivity analysis was conducted for confounding effect of the enhanced fluoroquinolone black box warning enacted in July 2016 by the US Food and Drug Administration (FDA), indicated here by the vertical dashed blue line. Grey shaded regions represent the staged intervention phases. Points represent actual antibiotic use in days of therapy per thousand patient days present. Dashed line represents modeled results from interrupted time series regression.

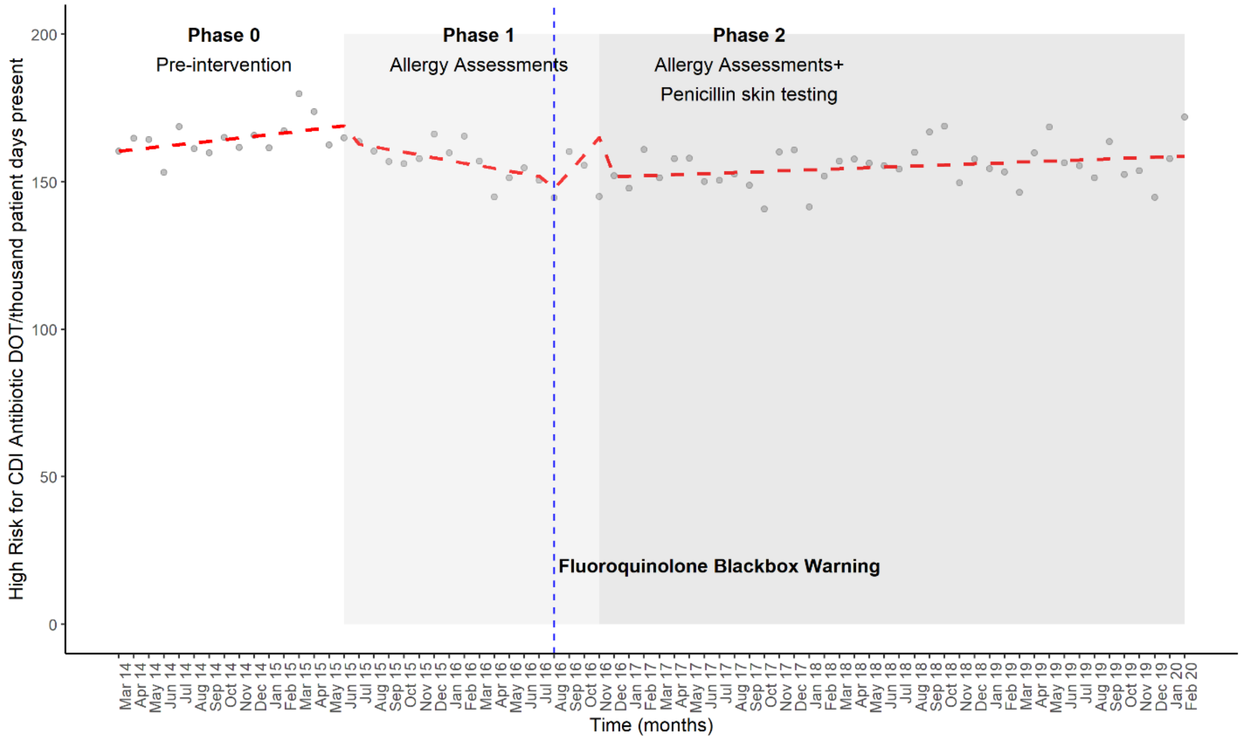

Model	Use Rate Ratio (95% CI)	p-value
**High risk for CDI antibiotics**		
Baseline time trend	1.04 (0.99-1.09)	0.10
Allergy assessment, level change	0.96 (0.91-1.02)	0.16
Allergy assessment, slope change	0.89 (0.82-0.97)	0.01
Penicillin skin test, level change	0.92 (0.82-1.03)	0.15
Penicillin skin test, slope change	0.65 (0.34-1.24)	0.19
Enhanced blackbox, level change	0.98 (0.90-1.06)	0.61
Enhanced blackbox, slope change	0.78 (0.87-3.18)	0.12

© 2021 Turner NA et al. *JAMA Network Open.*

eFigure 2. Interrupted time series analysis by individual antibiotic classes.

Sub-analysis of antibiotic use trends according to individual antibiotic classifications per table S1. Grey shaded regions represent the staged intervention phases. Points represent actual antibiotic use in days of therapy per thousand patient days present. Dashed lines represent modeled results from interrupted time series regression. Antibiotic class is delineated by line/point color.

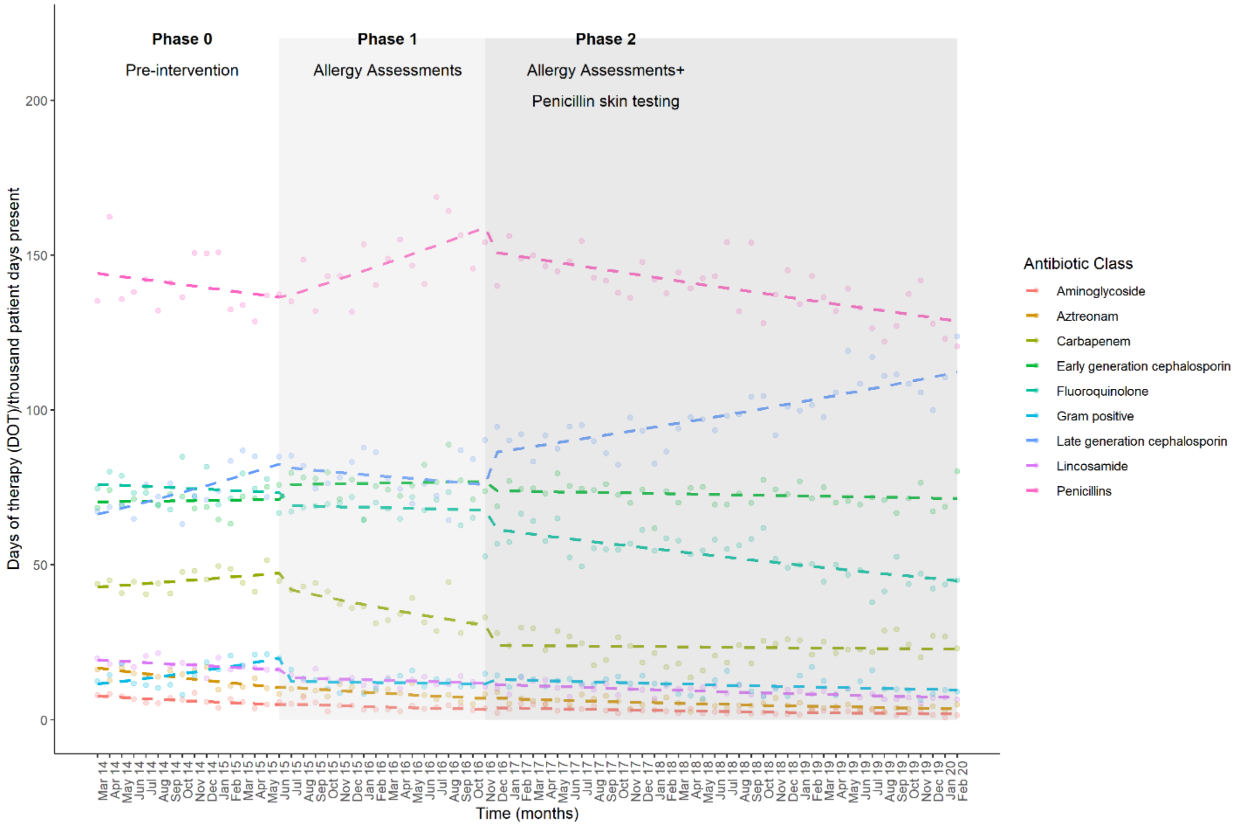

Model	Use Rate Ratio (95% CI)	p-value
**Aminoglycosides**		
Baseline time trend	0.69 (0.51-0.94)	0.02
Allergy assessment, level change	1.05 (0.75-1.46)	0.80
Allergy assessment, slope change	1.07 (0.69-1.64)	0.77
Penicillin skin test, level change	1.19 (0.88-1.60)	0.25
Penicillin skin test, slope change	1.06 (0.78-1.45)	0.72
**Aztreonam**		
Baseline time trend	0.70 (0.53-0.89)	<0.01
Allergy assessment, level change	1.03 (0.78-1.35)	0.86
Allergy assessment, slope change	1.08 (0.73-1.53)	0.77
Penicillin skin test, level change	1.04 (0.81-1.32)	0.47
Penicillin skin test, slope change	1.11 (0.84-1.46)	0.46

© 2021 Turner NA et al. *JAMA Network Open.*

Model (cont'd)	Use Rate Ratio (95% CI)	p-value
**Carbapenems**		
Baseline time trend	1.08 (0.89-1.31)	0.42
Allergy assessment, level change	0.88 (0.72-1.09)	0.23
Allergy assessment, slope change	0.73 (0.56-0.95)	0.02
Penicillin skin test, level change	0.79 (0.66-0.94)	0.01
Penicillin skin test, slope change	1.25 (1.03-1.52)	0.03
**Fluoroquinolones**		
Baseline time trend	0.97 (0.89-1.07)	0.54
Allergy assessment, level change	0.95 (0.85-1.05)	0.27
Allergy assessment, slope change	1.01 (0.89-1.16)	0.84
Penicillin skin test, level change	0.91 (0.84-0.99)	0.04
Penicillin skin test, slope change	0.92 (0.84-1.01)	0.09
**Early Generation Cephalosporins**		
Baseline time trend	1.01 (0.94-1.08)	0.82
Allergy assessment, level change	1.07 (0.99-1.15)	0.09
Allergy assessment, slope change	1.00 (0.91-1.10)	0.98
Penicillin skin test, level change	0.96 (0.90-1.03)	0.24
Penicillin skin test, slope change	0.98 (0.91-1.05)	0.57
**Late Generation Cephalosporins**		
Baseline time trend	1.19 (1.09-1.30)	<0.01
Allergy assessment, level change	0.97 (0.88-1.07)	0.53
Allergy assessment, slope change	0.80 (0.78-0.90)	<0.01
Penicillin skin test, level change	1.13 (1.04-1.22)	<0.01
Penicillin skin test, slope change	1.14 (1.05-1.25)	<0.01
**Lincosamide (Clindamycin)**		
Baseline time trend	0.87 (0.74-1.02)	0.09
Allergy assessment, level change	0.85 (0.71-1.02)	0.09
Allergy assessment, slope change	1.03 (0.82-1.31)	0.78
Penicillin skin test, level change	0.98 (0.84-1.15)	0.80
Penicillin skin test, slope change	0.96 (0.81-1.14)	0.67
**Gram positive agents**		
Baseline time trend	1.54 (1.19-2.00)	<0.01
Allergy assessment, level change	0.60 (0.45-0.81)	<0.01
Allergy assessment, slope change	0.61 (0.42-0.89)	0.01
Penicillin skin test, level change	1.13 (0.88-1.46)	0.34
Penicillin skin test, slope change	0.96 (0.73-1.26)	0.76
**Penicillins**		
Baseline time trend	0.96 (0.90-1.02)	0.19
Allergy assessment, level change	1.01 (0.94-1.08)	0.80
Allergy assessment, slope change	1.17 (1.06-1.28)	<0.01
Penicillin skin test, level change	0.95 (0.90-1.01)	0.12
Penicillin skin test, slope change	0.85 (0.80-0.91)	<0.01

© 2021 Turner NA et al. *JAMA Network Open.*

eTable 4. Interrupted time series analysis of hospital-associated *C. difficile* rates – Effect Estimates Accompanying Figure 2

Model	Incident Rate Ratio (95% CI)	p-value
**Hospital-associated CDI**		
Pre-intervention	1.85 (0.44-7.81)	0.40
Phase 1, level change	0.82 (0.49-1.37)	0.45
Phase 1, slope change	0.79 (0.18-3.44)	0.76
Phase 2, level change	0.35 (0.94-1.42)	0.58
Phase 2, slope change	0.61 (0.43-0.86)	<0.01

© 2021 Turner NA et al. *JAMA Network Open.*

eFigure 3. Sensitivity Analysis for Potential Outlier Effect of October 2016-December 2016 HA-CDI Rates

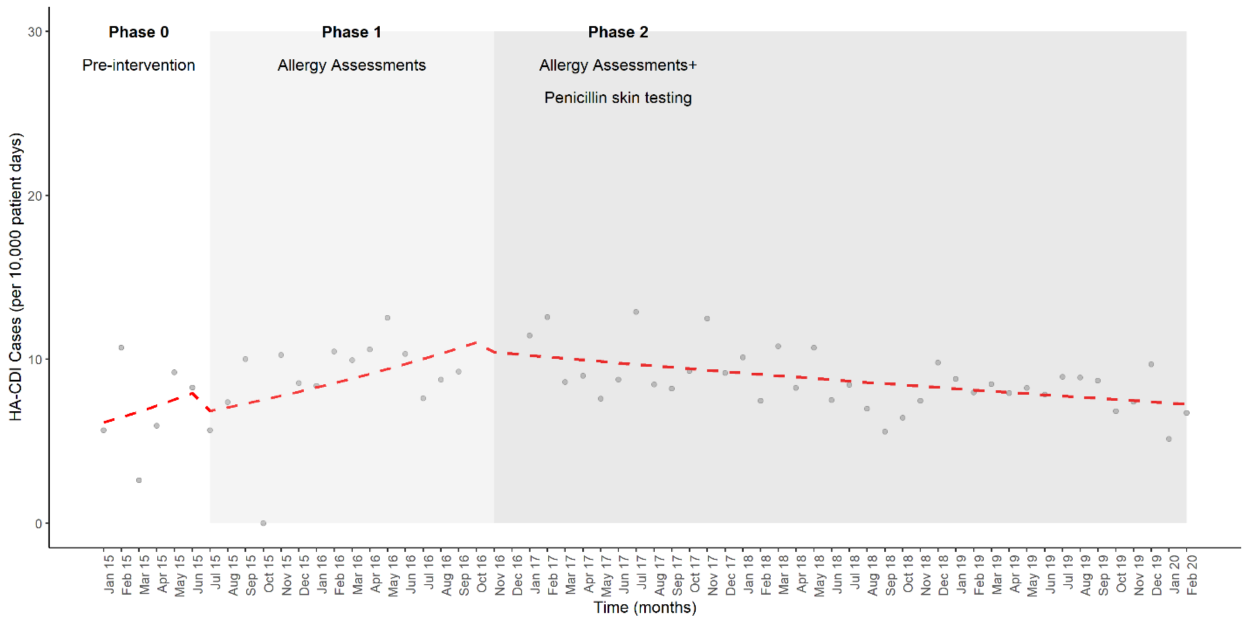

Model	Incident Rate Ratio (95% CI)	p-value
**Hospital-associated CDI**		
Pre-intervention	1.05 (0.93-1.19)	0.40
Phase 1, level change	0.82 (0.49-1.37)	0.45
Phase 1, slope change	0.77 (0.87-1.11)	0.76
Phase 2, level change	0.92 (0.68-1.24)	0.58
Phase 2, slope change	0.96 (0.93-0.99)	0.01

© 2021 Turner NA et al. *JAMA Network Open.*

eFigure 4. Distribution of Propensity Scores*

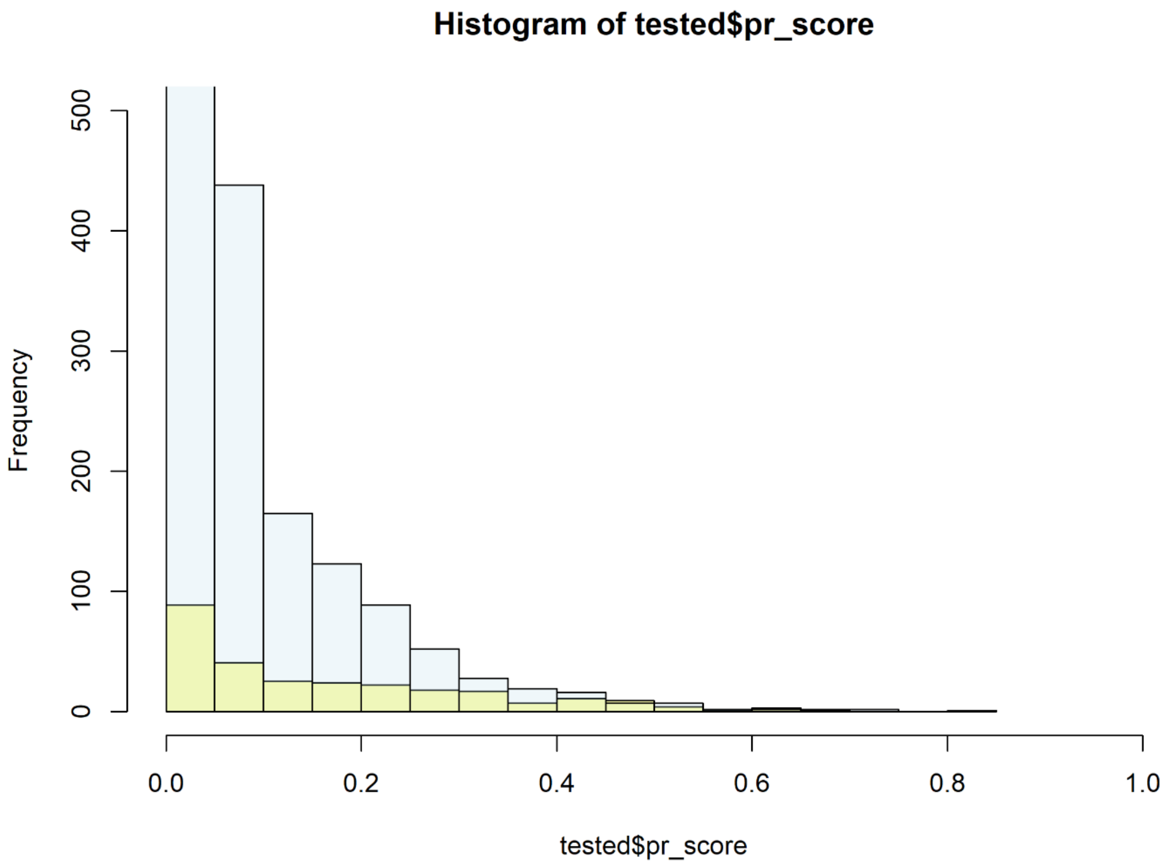

*Green represents propensity score distribution for penicillin skin tested subjects; blue represents propensity score distribution for untested subjects.

© 2021 Turner NA et al. *JAMA Network Open.*

eTable 5. Inpatient and Discharge Antibiotic Selection Analyzed by Penicillin Allergy Assessment Status

Antibiotics Received	Unevaluated, n=819 (%)	Evaluated, n=272 (%)	OR, evaluated vs unevaluated (95% CI)	P value
**Inpatient**				
Beta lactam	140 (17.1)	195 (71.7)	12.3 (9.0-17.0)	<0.01
Non-beta lactam	645 (78.8)	237 (87.1)	1.83 (1.25-2.74)	<0.01
High risk antibiotic	529 (64.6)	194 (71.3)	1.36 (1.01-1.85)	0.04
Aminoglycoside	17 (2.1)	5 (1.8)	0.88 (0.29-2.26)	0.81
Fluoroquinolone	307 (37.5)	89 (32.7)	0.81 (0.61-1.08)	0.16
Early gen cephalosporin	152 (18.6)	68 (25.0)	1.46 (1.05-2.02)	0.02
Late gen cephalosporin	328 (40.0)	119 (43.8)	1.16 (0.88-1.54)	0.28
Carbapenem	85 (10.4)	28 (10.3)	0.99 (0.62-1.54)	0.97
Lincosamide	154 (18.8)	52 (19.1)	1.02 (0.71-1.44)	0.91
Monobactam	93 (11.4)	66 (24.3)	2.50 (1.76-3.55)	<0.01
Gram Positive Agents	472 (57.6)	186 (68.4)	1.59 (1.19-2.13)	<0.01
Macrolide	62 (7.6)	18 (6.6)	0.87 (0.49-1.46)	0.60
Sulfa	97 (11.8)	31 (11.4)	0.96 (0.61-1.46)	0.84
Tetracyclines	46 (5.6)	21 (7.7	1.41 (0.81-2.37)	0.21
Other	14 (1.7)	6 (2.2)	1.30 (0.46-3.27)	0.60
**Upon discharge**				
Beta lactam	16 (2.0)	51 (18.8)	11.6 (6.62-21.33)	<0.01
Non-beta lactam	282 (34.4)	125 (46.0)	1.62 (1.22-2.14)	<0.01
High risk antibiotic	110 (36.9)	49 (27.8)	0.66 (0.44-0.98)	0.04
Aminoglycoside	0 (0.0)	0 (0.0)	--	--
Fluoroquinolone	71 (8.7)	32 (11.8)	1.40 (0.89-2.17)	0.13
Early gen cephalosporin	42 (5.1)	26 (9.6)	1.96 (1.16-3.23)	<0.01
Late gen cephalosporin	31 (3.8)	16 (5.9)	1.59 (0.84-2.91)	0.14
Carbapenem	14 (1.7)	4 (1.5)	0.86 (0.24-2.42)	0.79
Lincosamide	8 (1.0)	1 (0.4)	0.37 (0.02-2.05)	0.36
Monobactam	1 (0.1)	1 (0.4)	3.02 (0.12-76.50)	0.44
Gram Positive Agents	36 (4.4)	11 (4.0)	0.92 (0.44-1.77)	0.81
Macrolide	2 (0.2)	1 (0.4)	1.51 (0.07-15.80)	0.74
Sulfa	21 (2.6)	14 (5.1)	2.06 (1.01-4.08)	0.04
Tetracyclines	26 (3.2)	5 (1.8)	0.57 (0.19-1.38)	0.26
Other	30 (3.7)	14 (5.1)	1.43 (0.72-2.68)	0.28

© 2021 Turner NA et al. *JAMA Network Open.*

eTable 6. Inpatient and Outpatient Days of Therapy by Antibiotic and Penicillin Skin Testing Status

Days of Therapy	Untested, n=819 (%)	Tested, n=272 (%)	P value
**Inpatient**			
Beta lactam	1.1 (3.3)	2.9 (5.2)	<0.01
Non-beta lactam	13.0 (16.5)	12.5 (13.0)	0.70
High risk antibiotic	6.0 (9.2)	5.0 (7.0)	0.11
Aminoglycoside	0.1 (0.6)	0.1 (0.8)	0.57
Fluoroquinolone	1.8 (4.1)	1.4 (4.2)	0.16
Early gen cephalosporin	0.8 (3.2)	1.0 (3.2)	0.23
Late gen cephalosporin	2.8 (5.6)	2.5 (4.5)	0.39
Carbapenem	0.8 (3.3)	0.6 (2.6)	0.26
Lincosamide	0.6 (2.3)	0.5 (1.6)	0.93
Monobactam	0.7 (2.6)	1.0 (2.3)	0.08
Gram Positive Agents	4.1 (7.1)	4.2 (5.8)	0.89
Macrolide	0.3 (1.8)	0.2 (1.0)	0.19
Sulfa	0.6 (2.5)	0.6 (2.6)	0.79
Tetracyclines	0.3 (1.9)	0.4 (2.3)	0.52
Other	0.1 (0.7)	0.1 (0.5)	0.63
**Outpatient**			
Beta lactam	0.6 (3.3)	4.4 (10.0)	<0.01
Non-beta lactam	31.1 (71.1)	20.4 (21.8)	0.75
High risk antibiotic	9.0 (25.1)	6.6 (17.5)	0.27
Aminoglycoside	0 (0)	0 (0)	--
Fluoroquinolone	5.2 (23.9)	3.4 (9.3)	0.33
Early gen cephalosporin	2.9 (9.0)	2.6 (8.4)	0.65
Late gen cephalosporin	2.0 (7.3)	1.9 (7.4)	0.80
Carbapenem	1.3 (6.5)	1.3 (13.9)	0.98
Lincosamide	0.4 (3.2)	0.1 (0.8)	0.14
Monobactam	0.1 (2.1)	2.2 (29.8)	0.22
Gram Positive Agents	4.0 (23.0)	1.8 (7.4)	0.22
Macrolide	1.2 (21.1)	0.0 (0.5)	0.45
Sulfa	3.7 (30.3)	7.2 (47.5)	0.33
Tetracyclines	31.1 (71.1)	20.4 (21.8)	0.75
Other	2.7 (22.0)	3.9 (28.8)	0.62
**Total duration of therapy**	9.3 (12.1)	9.8 (11.4)	0.62

*Mean (SD)

© 2021 Turner NA et al. *JAMA Network Open.*

eTable 7. Cox Proportional hazard Estimates

A, Overall survival, Stratified by Penicillin Allergy Assessment (accompanying figure 4A)

	Cox Proportional Hazard Modeling of Mortality					
	Overall Cohort			Matched Cohort		
	Unassessed	Assessed	HR (95% CI)	Unassessed	Assessed	HR (95% CI)
Survival	2,329/11,308 (20.6)	45/273 (16.5)	0.86 (0.64-1.16)	225/819 (27.5)	44/272 (16.2)	0.77 (0.55-1.07)

B, Stratified by Penicillin Allergy Assessment (accompanying figure 4B)

	Cox Proportional Hazard Modeling of HCFA-CDI Risk					
	Overall Cohort			Matched Cohort		
	Unassessed	Assessed	HR (95% CI)	Unassessed	Assessed	HR (95% CI)
HCFA CDI Free Survival	111/11,308 (1.0)	4/273 (1.5)	1.47 (0.54-4.00)	22/819 (2.7)	4/272 (1.5)	0.53 (0.18-1.55)

© 2021 Turner NA et al. *JAMA Network Open.*
